# Association between choices of transportation means and instrumental activities of daily living: observational cohort study of community-dwelling older adults

**DOI:** 10.1186/s12889-022-14671-y

**Published:** 2023-01-26

**Authors:** Motoki Tamura, Ishikawa Tomoki, Komaki Matsumoto, Shinji Hattori

**Affiliations:** 1 Institute for Health Economics and Policy, Tokyu Toranomon Bldg, 1-21-19 Toranomon, Minato-ku, Tokyo 105-0001 Japan; 2 Chiba University Center for Preventive Medicine, 1-33, Yayoicho, Inage-ku, Chiba Prefecture Chiba, 263-8522 Japan; 3 Toyoake City Office, Citizens Collaboration Div., 1-1 Komochimatsu, Shindencho, Toyoake, Aichi Prefecture 470-1195 Japan

**Keywords:** Choice of transportation means, Instrumental activities of daily living, Active means of transportation, Passive means of transportation, Long-term care prevention

## Abstract

**Introduction:**

The association between the physical health of older people and the frequency of going out has been reported, and in recent years, local governments have developed transportation support programs for older people. Although previous studies show an association between the frequency of going out and functional health status, little has been reported on the impact of the choice of means of transport on instrumental activities of daily living (IADL).

**Objective:**

To evaluate the association between choice of transportation means and the risk of decline in IADL among older adults.

**Methods:**

We conducted an observational, population (community-dwelling)-based cohort study using data from the Resident Health Status Survey, and longitudinal panel data at 2-time points in 2016 and 2019. In addition, we combined this panel data and a database on people who were certified as requiring long-term care to identify participants’ IADL. The propensity score matching method was used to classify the respondents into two groups, “active means of transportation” and “passive means of transportation,“ and determine the risk of a decline in means-tested independence after 3 years.

**Results:**

Active means were used by 6,280 (76.2%) and passive means were used by 1,865 (22.6%). 999 (12.1%) individuals declined in IADL in 2019. The results of the comparison by balancing the attributes of “active means of transportation” and “passive means of transportation,“ with propensity score matching, showed that “passive means of transportation” were more likely to be “active” than “passive means of transportation,“ and “active” was more likely to be “passive” The risk of IADL decline was significantly higher than that of “active means of transportation” with an RR of 1.93 (95% CI: 1.62–2.30).

**Conclusion:**

Passive means of transportation in older adults could be a possible risk for decreasing IADL 3 years later. Increasing the number of opportunities and places in the community for older adults to use active means of transportation may be effective in encouraging socially independent living among older adults.

## Introduction

The maintenance of instrumental activities of daily living (IADL) level is one of the major concerns in community policy and an essential factor for older adults to live independently and healthily. Many local governments have an increasing number of older adults and face the social demand to develop effective countermeasures. Previous studies report that PA is mediated by functional limitations and affects the impairment of ADL/IADL [1]. IADLs represent the behavioral abilities most relevant for older adults to live independently, ranging from general household activities such as cleaning and laundry to more applied activities such as money management, internal medicine management and use of public transport [2–5]. And declining IADL is a predictor of cognitive decline and institutionalization [2–4]. Therefore, the management IADL of in older adults would contribute prevention of declining their PA, and public demand for environmental construction to implement one seems to be growing in coming years.

According to the World Health Organization (WHO), between 2015 and 2050, the proportion of the world’s population aged 60 years and older will increase from 12–22% [5]. In Japan, the population aged 65 and over is estimated to grow from 26.6% to 2015 to 38.1% in 2060 [6]. Because the number of older adults is expected to increase, it will become even more essential to maintain IADL among older adults. Especially in Japan, currently, the oldest country globally, encouraging daily activities to reduce the risk of declining IADL has attracted public attention.

The association between transportation and health has received particular interest from the perspective of administrative mobility support in recent years. Previous studies have found that mobility for older adults is associated with health, including lower depressive symptoms (mental) [7], better maintenance of PA and lower BMI (physical) [8–10], and fostering social capital (social health) [11]. However, some studies already have reported that ensuring a means of transportation for older adults is expected to maintain or improve their functional health, but little has been reported on IADLs.

In addition, a longitudinal cohort study has reported the association between types of transportation and health outcome, such as functional limitations and requiring long-term care, but these limited the target population to frail older adults so that whether similar findings are generalizable to older adults in the community has not been evaluated [12]. Moreover, the difference of effect over time by the type of transportation on IADL has not been clarified, since almost all previous studies are cross-sectional designs [13, 14]. Understanding the association between the difference of effect over time and the type of transportation could provide useful knowledge for care prevention.

Using 3-years of longitudinal public survey data in Japan, this study was performed to (i) examine the association between the means of transportation and IADLs among community-dwelling older adults and (ii) clarify whether the risk of a decline in IADLs differs between older adults who use different means of transportation.

## Materials and methods

### Data source

We conducted a retrospective, observational, population (community-dwelling)-based cohort study using data from the Resident Health Status Survey (Long-Term Care Prevention and Daily Life Area Needs Survey) conducted by Toyoake City, Aichi Prefecture, Japan [15–17]. This survey was conducted by a long-term care insurer to contribute to community diagnosis in different areas of daily living for community residents aged 65 years and older eligible for long-term care services or preventive care services [18]. Thus, we used data that were collected from participants in a survey of community-dwelling adults aged 65 years or older who were not certified as requiring long-term care. Participation in the survey was voluntary, and we considered those who returned the survey questionnaire as potential participants in this survey. No personally identifiable information was included in the data used for our analysis. This study followed the Strengthening the Reporting of Observational Studies in Epidemiology (STROBE) reporting guidelines for observational studies [19].

### Study participants

**Fig. 1:**
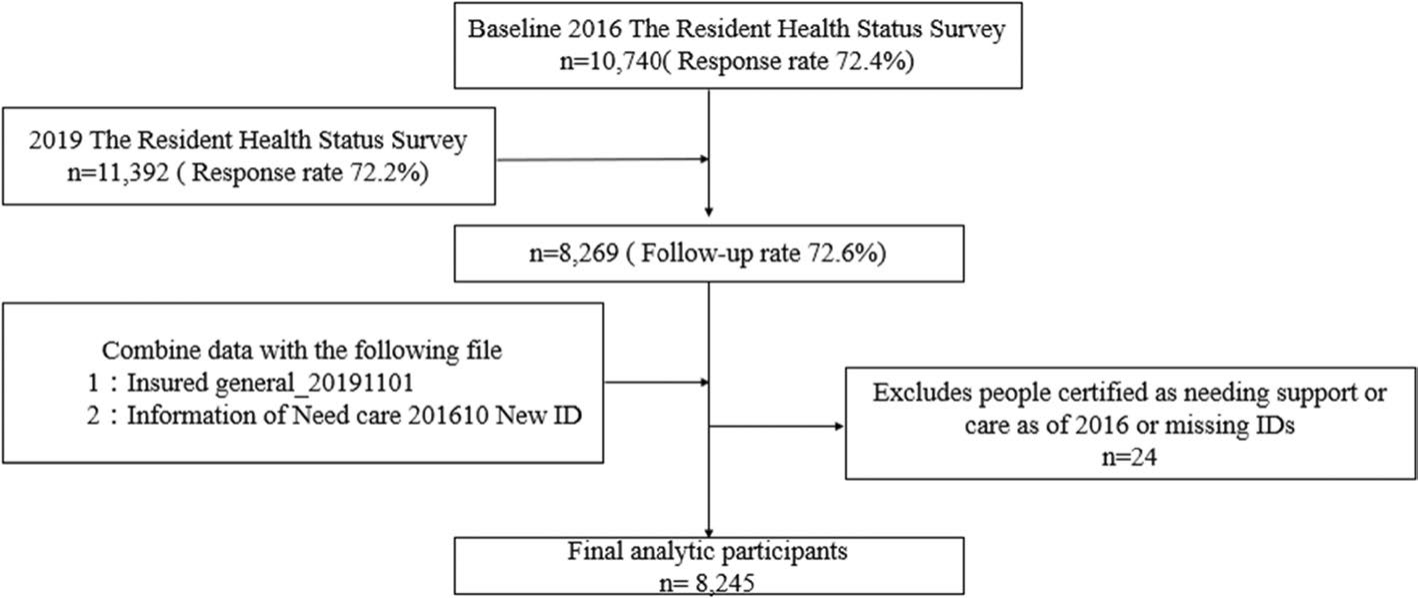
Flow chart of the participants selection

Figure 1 shows the flowchart for the identification of exposed/unexposed (below for definition) cohort with the eligibility/exclusion criteria. For the baseline survey, the questionnaire was distributed by mail to 14,844 people, of whom 10,740 (72.4% response rate) responded. Of these, 8,269 (72.6% matching rate) were also able to be tracked in the follow-up survey.

The data for individuals who were certified as requiring long-term care in October 2016 and November 2019 were combined, and the baseline survey. We excluded 24 participants who met one or more of the exclusion criteria, including missing ID or requiring long-term care at baseline. After all exclusions, 8,245 respondents were included in our analyses.

### Participants selection

We utilized this survey data as panel data for two-time points: November 2016 (as the baseline survey) and November 2019 (as the follow-up). Figure 1 shows the flowchart for the identification of exposed/unexposed (below for definition) cohort with the eligibility/exclusion criteria. For the baseline survey, the questionnaire was distributed by mail to 14,844 people, of whom 10,740 (72.4% response rate) responded. Of these, 8,269 (72.6% follow-up completed) were also able to be tracked in the follow-up survey. To identify individuals who have not been certified as requiring long-term care before the baseline survey, the historical data of long-term care certification information in October 2016 and November 2019 were merged into the baseline survey. 24 participants were excluded by exclusion criteria: missing ID, non-response on baseline survey, or already required long-term care at baseline.

### Outcome variable (IADLs)

We set the status of whether or not the participant had achieved IADLs as the outcome variable. IADLs were measured using the five-item Tokyo Metropolitan Institute of Gerontology Index of Competence (TMIG-IC), which is based on the Lawton IADL scale [20, 21]:(i) going out alone by bus or train, (ii) shopping for food and daily necessities by oneself, (iii) preparing meals by oneself, (iv) paying bills by oneself and (v) withdrawing money from savings by oneself.

Responses to these items were classified into two categories, “I can and do,” and “I can not,” and the results of totaling the relevant items with one point per item were defined as the index of IADL.

There was a score of 5 was defined as a condition of “independent” and a score of 4 or lower was defined as a “dependent” [22, 23]. The outcome variable was the incidence of loss of IADL (less than 5 points) in 2019.

### Exposure variable (Means of transportation)

The data from the survey were used to divide each participant’s exposure status as active or passive in terms of daily transportation. In the questionnaire item “Means of transportation when going out,” “walking,” “senior car,” “bicycle,” “motorcycle,” “car (driving by oneself),” “train,” and “local bus” were collectively defined as “active means of transportation”. “Cars (driven by others)” and “cabs” were defined as “passive means of transportation” and were set as exposure variables. In this study, the group of self-reported responses that using only the active means of transportation in this study was defined as “active group”, and those using any of the passive means of transportation were defined as “passive group”.

### Covariates

To control for relevant potential confounders, we set the following variables as covariates for adjustment based on the baseline survey data. Each covariate has been reported to be associated with both outcome and exposure variables in previous studies [7, 9, 10].

These covariates included participants’ age(65–69, 70–74, 75–79, 80–84, 85 years and older); sex; socio-economic status (SES), such as family structure and subjective economic poverty; health status and health behaviors; smoking; Body mass index (BMI); cognitive decline; and Number of chronic diseases (0,1,2,3 or more); loss of IADL (less than 5 points)in 2016. Cognitive decline was assessed using a questionnaire (called the Kihon Checklist in Japan) [24]. The predictive validity of the basic checklist has already been verified [25]. Chronic diseases were calculated based on self-rated diseases (hypertension, stroke, heart disease, diabetes, hyperlipidemia, respiratory disease, gastrointestinal and liver disease, kidney disease, musculoskeletal disease, trauma, cancer, blood disease, depression, dementia, Parkinson’s disease, eye disease, and ear disease).

### Statistical analysis

Missing values were completed by multiple imputation by chain equation (MICE) before propensity score analysis was conducted.

First, we compared baseline characteristics of participants by active or passive means of transportation exposure status for categorical variables. Next, a matching method using propensity scores was conducted to compare functional and attribute-matched passive means of transportation. Estimates of the propensity scores of the subjects were obtained by logistic regression analysis with active or passive means of mobility as the objective variable and the covariates as explanatory variables.

The algorithm used nearest neighbor matching to randomly select one person from the participant group and pair the propensity score of the chosen participant with the person with the closest propensity score from the non-participant group. Calipers were matched to those who fell within a certain propensity score threshold (caliper) using the specification method. The caliper was defined as the standard deviation of the logit-transformed value of the estimated propensity score multiplied by 0.2 [26].

One participant and one non-participant were matched by pair matching with a composition ratio of 1:1. Non-reciprocal sampling was used, whereby the same non-participant could not be used more than once as a pair in the participation group. Standardized differences were calculated to check the balance between the groups after matching. The standardized difference is an index of the degree of balance between the active and passive mobility groups and was judged to be balanced if it was less than 0.1 [27]. After the balance was confirmed, Poisson regression analysis was performed to determine the risk ratio (RR). The objective variable was whether or not IADL decline was observed in 2019 and the explanatory variables were active and passive means of transportation.

In addition, as a sensitivity analysis, we alternatively defined IADL criteria by replacing “I can do but have not done” with “I cannot do”, to assess the robustness by the handling of intermediate answers where the answers differ depending on the assumptions of the respondent. The significance level was set at 5%, and STATA 16/SE (Stata Corp LLC, College Station, TX) was used.

## Results

### Attributes of participants

Table 1 shows the characteristics of the subjects in the 2016 survey and the standardized differences before and after matching. The distribution of propensity scores before and after matching is also shown in Figs. 1 and 2 of the supplemental.

Of the 8,245 subjects analyzed (mean age ± standard deviation, 73.2 ± 5.7), 3,823 were men and 4,422 were women. There were 6,280 (77.1%) people with active means of transportation and 1,865 (22.9%) with passive means of transportation. In 2019, 999 (12.1%) people exhibited a decline in IADL (less than 5 points), 727 (11.6%) people had an active means of transportation, and 255 (13.7%) people had a passive means of transportation. Participants with active means of transportation were more likely to be men, to be 65–69 years old, to live together with their spouse as a married couple(spouse under 65 years old), to have a normal level of subjective economic poverty, to have a BMI ≥ 18.5–<25, to everyday smoke, to have cognitive decline, to, and to have chronic diseases.

**Fig. 2:**
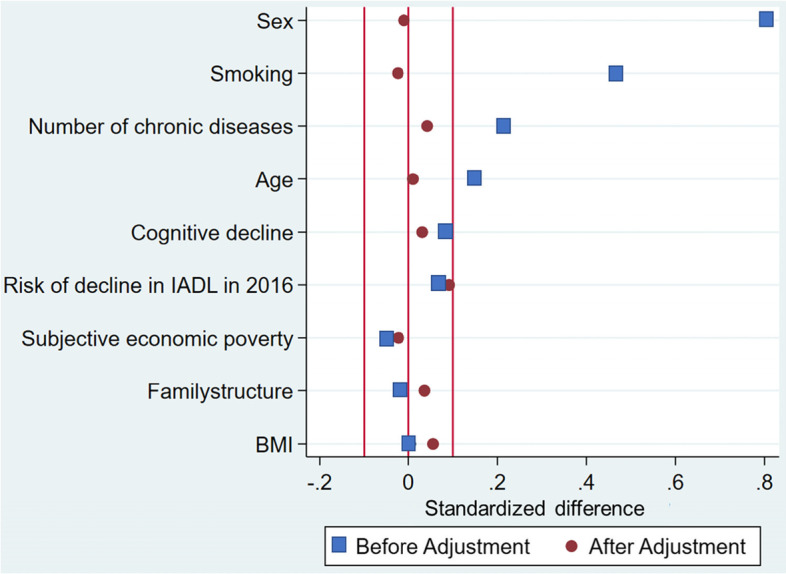
The standardized difference for baseline covariates in the original and the matched groups

In contrast, participants with passive mobility were more likely to be women, to be 70–74 years old, to live together with their spouse as a married couple (spouse under 65 years old), to have a normal level of subjective economic poverty, to have a BMI ≥ 18.5–<25, to everyday smoke, to have cognitive decline, to, and to have chronic diseases. Standardized differences after matching shown in Fig. 2 by matching were less than 0.1 for all covariates.

**Table 1:** Overall socioeconomic status and health status of subjects by means of transportation

		Total	The active of mean transportation	The passive of mean transportation
		*n* = 8,245	*n* = 6,280(%)	*n* = 1,865(%)
Decline in IADL in 2016 (<5 points)*	yes	945(11.46)	712(11.34)	215(11.53)
Decline in IADL in 2019 (<5 points)*	yes	999(12.12)	727(11.58)	255(13.67)
Gender	Female	4,422(53.63)	2,814(44.81)	1,558(83.54)
Age,yr	65–69	2,688(32.6)	2,156(34.33)	504(27.02)
	70–74	2,425(29.41)	1,871(29.79)	528(28.31)
	75–79	1,902(23.07)	1,430(22.77)	451(24.18)
	80–84	927(11.24)	649(10.33)	262(14.05)
	≧ 85	303(3.67)	174(2.77)	120(6.43)
Family structure	Married couple living together (spouse 65 years or older)	977(11.85)	786(12.52)	189(10.13)
	Married couple living together (spouse under 65 years old)	3,526(42.77)	2,650(42.2)	865(46.38)
	Two households with son or daughter	477(5.79)	420(6.69)	56(3)
	living alone	1,591(19.3)	1,198(19.08)	384(20.59)
	Other	1,474(17.88)	1,127(17.95)	340(18.23)
Subjective economic poverty	Very leeway	457(5.54)	335(5.33)	116(6.22)
	Leeway	1,737(21.07)	1,341(21.35)	387(20.75)
	Normal	5,286(64.11)	4,054(64.55)	1216(65.2)
	Slightly hard	551(6.68)	446(7.1)	104(5.58)
	Very hard	78(0.95)	58(0.92)	20(1.07)
BMI	< 18.5	5,900(71.56)	4,586(73.03)	1,295(69.44)
	≥ 18.5-<25	830(10.07)	526(8.38)	229(12.28)
	≥ 25-<30	1,386(16.81)	1,080(17.2)	302(16.19)
	≥ 30-<50	129(1.56)	88(1.4)	39(2.09)
Smoking	None	664(8.05)	589(9.38)	68(3.65)
	Past	95(1.15)	75(1.19)	19(1.02)
	Sometime	2,391(29)	2,143(34.12)	219(11.74)
	Every day	4,965(60.22)	3,390(53.98)	1,528(81.93)
Cognitive decline	Yes	4,773(57.89)	3,700(58.92)	1,015(54.42)
Number of chronic diseases	0	327(3.97)	238(3.79)	72(3.86)
	1	4,327(52.48)	3,413(54.35)	869(46.6)
	2	2,008(24.35)	1,530(24.36)	459(24.61)
	3+	1,583(19.2)	1,099(17.5)	465(24.93)

^*^There was a score of 5 was defined as a condition of “independent” and a score of 4 or lower was defined as a “dependent”

### Transportation and IADL

Table 2 shows the results of the Poisson regression analysis with the risk of a decline in IADLs in 2019 as the outcome variable. The estimation results of propensity score matching showed that passive means of transportation were associated with a significantly higher risk of a decline in IADLs than active means of transportation (RR: 1.93; 95% CI: 1.62–2.30). The results of sensitivity analysis showed that passive means of transportation were associated with a significantly higher risk of a decline in IADLs compared with active means of transportation (RR: 1.35; 95% CI, 1.25–1.45).

**Table 2:** Association between means of transportation and risk of loss of IADL

	RR^a^	*P*-value	95%CI^b^
Active means of transportation^d^	1.00		
Passive means of transportation^d^	1.93^c^	< 0.001	(1.62–2.30)

^a^Risk ratio

^b^*CI* Confidence interval

^c^Results of adjusting for different background factors by propensity score matching

^d^Subjects after PS matching：Active means of transportation *N*= 2,778, Passive means of transportation *N*= 1,786

## Discussion

In the current study, we compared the risk of a decline in IADLs among older adults who used either active or passive means of transportation. The results showed that the risk of decline in IADLs after 3 years was 1.93 times higher for older adults who used passive means of transportation than for those who used active means of transportation. Thus, the results suggest that older adults who can transport themselves have a reduced risk of a decrease in the higher-order activity skills necessary for socially independent living. Therefore, ensuring older adults have access to active means of transportation may promote “healthy aging” by reducing the risk of decreases in the higher-order activity skills needed to lead a socially independent life.

Our findings support those of previous studies in Japan, that found differences in mean IADL scores among older adults according to their choice of means of transportation [13]. Furthermore, car and public transportation users have been shown to be go out more frequently [14, 28, 29], and the transportation to driving cessation is associated with functional limitations for the older adults [30]. Therefore, active means of transportation may maintain a higher frequency of going out than passive means of transportation, thus lowering the risk of IADL decline.

Active means of transportation, as defined in this study, may promote opportunities to going out and walk [8, 9] and maintain and improve PA as older adults travel to destinations, including train stations and bus stops, as a decline in PA is mediated by functional limitations and affects impairment of instrumental activities of daily living (ADLs/IADLs). Since PA decline has already been reported to affect impairments in instrumental activities of daily living (ADL/IADL), mediated by functional limitations [1], active means of transportation that are expected to maintain and improve PA and passive means of transportation without it might be the difference in worsening IADLs. This could partly explain why IADLs worsening was lower for active mobility measures than for passive mobility measures. Furthermore, compared to passive means of transportation, social life with active means of transportation is more likely to be performed with components of IADLs: (i) going out alone by bus or train, (ii) shopping for food and daily necessities by oneself, (iv) paying bills by oneself and (v) withdrawing money from savings by oneself. For example, in active means of transportation, a person can travel alone on public transport, shop, pay bills at the bank and withdraw money from savings. On the other hand, passive mobility leads to leaving the mobility to others, which may make it difficult for a person to continue daily life alone. These mechanisms may explain the higher deterioration in IADLs with passive means of transportation compared to active means of transportation.

Loss of IADL is widely known to be one of the risk factors for dementia [1]. Maintaining and promoting active mobility may have significant care prevention benefits for the older population if local governments introduce policies to maintain and promote active transportation. Previous studies that have clarified transportation and risk of long-term care needs have also analyzed frail older adults in need of care [12]. The key finding of this study is to identify older adults who were strictly independent at baseline and to show that different means of transportation can predict the risk of IADL decline at 3 years.

We performed a sensitivity analysis to examine the robustness of our results. The sensitivity analysis showed 1.35 times higher risk of a decline in IADLs for passive transportation compared to active one, and this result estimated lower the risk than the main analysis. Therefore, we confirmed the estimation result, that the risk of a decline in IADLs for passive transportation would lower, was consistent not depend on handling intermediate answer.

The novelty of the present study is that is the first to identify an association between the risk of loss of capacity for the necessary high-level activities of socially independent living and the means of transportation over time. Previous cross-sectional studies have reported that there is an association between means of transport and IADLs [13]. In addition, it has been shown that means of transportation, such as public transport, are associated with PA, but it was not clear whether different means of transportation were associated with the risk of IADL loss. In contrast, the results of this study suggest that the environment and individual choice available for active means of transportation are essential, which is an important finding.

A strength of the current study is that it included longitudinal analysis of panel data from two-time points for people aged 65 or older. Toyoake City, the target of the current study, has implemented transportation support measures for older adults, and these measures may be associated with the maintenance of the ability to perform higher-order activities that are required for a socially independent life. Considering community public health policies, measures to support transportation for older adults before they develop a condition requiring long-term care may have important social implications. In the current study, we identified the community-dwelling population through the linkage of multiple administrative survey datasets. This allowed us to analyze older adults before they develop a condition requiring long-term care, contributing to the improvement of internal validity. Finally, the background factors of the study subjects were aligned at baseline using a propensity score matching method to predict the risk of a decline in IADLs at 3 years. Previous studies may have included frail older adults at baseline [13]. This study combined with other administrative data to strictly limit the target population to independent older adults at the baseline survey. This is a strength of this study in that it estimated the risk of a decline in IADLs after 3 years for different means of transportation.

However, the current study involved several limitations that should be considered. First, the frequency of transportation use was not studied based on the questionnaire used in this study. Therefore, we were not able to assess the association between the amount of exposure and the outcome. In the future, it would be useful to investigate the frequency of the means of transportation. Second, the survey data used in this study were 3-year follow-up data, therefore, respondents’ status before 2016 is not known. It should be noted that we were not able to conclude long-term changes because we only observed short-term changes. In the future, pre-baseline adjustments and estimations that better eliminate the possibility of reverse causation, such as using three-point panel data, will be necessary. Third, we were not able to adjust for items not measured in this study, including unmeasured potential confounders. This is a limitation of observational studies using survey data held by municipal governments, and measurements related to instrumental self-reliance, such as household income and individual social roles, are currently lacking. Although we tried to minimize the influence of unmeasured confounders (e.g., using the subjective assessment of economic poverty as an alternative indicator for household income), we plan to conduct analyses based on actual measurements in a future study. Finally, the data from the present study do not contain information on the frequency of use of active and passive means of transport. Therefore, in this study, we cannot discuss the relationship between the frequency of use of each transportation and the risk of decreased activities of daily living. For example, it is not possible to determine from our data whether passive means of transport are used daily or occasionally. The association between the frequency of use of each means of transportation and IADLs is required to be discussed in future studies. Finally, the two groups of means of transportation are up for discussion. In future research, the groupings could be defined more strictly.

## Conclusion

In the current study, transportation was associated with the risk of IADL decline among community-dwelling older adults. Their use of passive means of transportation was shown to be a possible risk for the decline of IADL after 3 years. Measures to encourage the use of active means of transportation, including walking and self-controlled, may be effective in preventing the need for nursing care in the daily lives of older adults. Increasing the number of opportunities and places in the community for older adults to use active means of transportation may be effective in encouraging socially independent living among older adults.

## Data Availability

The datasets generated and analyzed during the current study are not publicly available due to the nature of the data. The dataset can be utilized for the limited use and its sharing with third parties is not allowed but needs the additional will be shared on request with the permission of the Toyoake City in Aichi prefecture, Japan.

## Abbreviations

BMI: Body Mass Index
IADL: Instrumental Activities of Daily Living
RR: Risk Ratio
MICE: Multiple Imputation by Chain Equation
PA: Physical Activity
SES: Socio-Economic Status
TMIG-IC: Tokyo Metropolitan Institute of Gerontology Index of Competence
